# Sterol-Derived Hormone(s) Controls Entry into Diapause in Caenorhabditis elegans by Consecutive Activation of DAF-12 and DAF-16

**DOI:** 10.1371/journal.pbio.0020280

**Published:** 2004-09-21

**Authors:** Vitali Matyash, Eugeni V Entchev, Fanny Mende, Michaela Wilsch-Bräuninger, Christoph Thiele, Arndt W Schmidt, Hans-Joachim Knölker, Samuel Ward, Teymuras V Kurzchalia

**Affiliations:** **1**Max Planck Institute for Molecular Cell Biology and GeneticsDresdenGermany; **2**Institute of Organic Chemistry, Technical University of DresdenDresdenGermany; **3**University of Arizona, TucsonArizonaUnited States of America

## Abstract

Upon starvation or overcrowding, Caenorhabditis elegans interrupts its reproductive cycle and forms a specialised larva called dauer (enduring). This process is regulated by TGF-β and insulin-signalling pathways and is connected with the control of life span through the insulin pathway components DAF-2 and DAF-16. We found that replacing cholesterol with its methylated metabolite lophenol induced worms to form dauer larvae in the presence of food and low population density. Our data indicate that methylated sterols do not actively induce the dauer formation but rather that the reproductive growth requires a cholesterol-derived hormone that cannot be produced from methylated sterols. Using the effect of lophenol on growth, we have partially purified activity, named gamravali, which promotes the reproduction. In addition, the effect of lophenol allowed us to determine the role of sterols during dauer larva formation and longevity. In the absence of gamravali, the nuclear hormone receptor DAF-12 is activated and thereby initiates the dauer formation program. Active DAF-12 triggers in neurons the nuclear import of DAF-16, a forkhead domain transcription factor that contributes to dauer differentiation. This hormonal control of DAF-16 activation is, however, independent of insulin signalling and has no influence on life span.

## Abstract 2

A sterol-derived activity is partially purified and shown to support reproductive growth under sterol-free conditions that normally induce dauer larva formation in nematodes

## Introduction

Sterols are essential in most eukaryotic cells and play a structural role in the architecture of their membranes. They influence the physicochemical properties of membranes, including fluidity and permeability for ions (Haines 2001). Cholesterol, together with glycosphingolipids, is also proposed to organise membrane microdomains (also called “rafts”), which provide platforms for protein sorting or signal transduction (Simons and Toomre 2000). In addition to this structural role in the membrane, cholesterol is essential for a variety of signalling processes. It is a precursor of important classes of physiologically active compounds such as steroid hormones in mammals or ecdysones in insects. The nematode Caenorhabditis elegans provides a valuable model system to study the orchestration of cholesterol metabolism and function at the level of a whole organism. *C. elegans,* like other nematodes, cannot synthesise sterols de novo (Hieb and Rothstein 1968; Chitwood 1999). Thus, it requires an exogenous source of sterols, which enables (i) analysis of sterol metabolism using labelled precursors and (ii) analysis of sterol functions by feeding normal and mutant worms with cholesterol derivatives and related sterols.

Although worms require exogenous cholesterol for survival, the effects of its depletion are still controversial (Kurzchalia and Ward 2003). Worms are routinely grown in the laboratory on agar plates seeded with bacteria and supplemented with 5 μg/ml of cholesterol (Brenner conditions) (Brenner 1974). Omitting sterols from agar has a weak effect on development and growth: Worms can still propagate for many generations, although some larvae fail to shed the old cuticles properly during molting, gonad development is aberrant, and movement is uncoordinated (Yochem et al. 1999; Shim et al. 2002). Under these conditions, the amounts of sterols in both the agar and the bacteria grown on yeast extracts seem to be sufficient to support growth. A stronger phenotype is obtained by using bacteria grown on defined or sterol-extracted media (Crowder et al. 2001; Merris et al. 2003). Results of depletion experiments indicate that although absolutely necessary, sterols are required only in very low amounts. This makes it less likely that they are structural components in worm membranes, and thus the primary role in worms should reside in signalling (Kurzchalia and Ward 2003). However, no specific signalling molecules derived from cholesterol, steroid hormones, or ecdysones have been identified yet.

It has been suggested that in worms cholesterol plays a role in the processes of molting and dauer formation. Involvement in molting is based on the roles of the worm homologues of mammalian megalin and insect DHR3. A worm mutant of *lrp-1,* a homologue of mammalian gp330/megalin protein, had a phenotype of defect in shedding of the cuticle, and this phenotype became more apparent upon partial cholesterol depletion (Yochem et al. 1999). Among other functions, megalin in mammals is involved in the uptake of a cholesterol derivative, vitamin D, by kidney absorptive cells (Willnow et al. 1999). Molting in insects is regulated by ecdysones, polyhydroxylated sterols derived from cholesterol, which act via nuclear hormone receptors. The analysis of the C. elegans genome did not reveal a homologue of the ecdysone receptor itself. However, disruption by RNAi of CHR3 *(nhr-23),* a C. elegans homologue of *Drosophila* orphan nuclear receptor (DHR3) that is induced by ecdysone, leads to defective shedding of the old cuticle (Kostrouchova et al. 1998, 2001).

Another process that might involve cholesterol or its derivatives is dauer larva formation. Many genes can mutate to cause constitutive formation of dauer larvae (Daf-c mutants) or to prevent their formation (Daf-d mutants) (Riddle and Albert 1997). Genetic studies have revealed that three pathways (TGF-β, cyclic GMP, and insulin-like IGF-1) control the formation of dauer larvae (Riddle and Albert 1997). DAF-2 (insulin-like receptor, IGF-1) signals to inhibit the activity of DAF-16, a forkhead domain (FOXO) transcription factor (Kenyon et al. 1993; Morris et al. 1996) that also influences the prolongation of adult life span (Lin et al. 1997; Ogg et al. 1997). Under dauer formation conditions, DAF-16 is activated and translocated into the nucleus, where it may integrate insulin-like and TGF-β signalling pathways (Henderson and Johnson 2001; Lee et al. 2001; Lin et al. 2001). Genetic epistasis analysis suggests that *daf-16* acts upstream of two other *daf* genes, *daf-9* and *daf-12* (Gerisch et al. 2001; Jia et al. 2002). It was proposed that DAF-16 inhibits the activity of DAF-9 when the dauer formation process is initiated. The integration of all three pathways downstream from *daf-9* occurs at the level of DAF-12, a putative nuclear hormone receptor (Antebi et al. 1998, 2000), suggesting a possible hormonal regulation of dauer larva formation. In addition, *daf-9* has a strong homology to several cytochrome P450s that are involved in steroid metabolism in mammals (Gerisch et al. 2001; Jia et al. 2002). The *daf-9* null mutation leads to constitutive dauer formation, consistent with the scenario where DAF-9 is an enzyme that produces a steroid hormone regulating DAF-12, which in turn ultimately triggers dauer formation.

As a starting point for our investigations on the role of sterols in *C. elegans,* we developed a protocol for strict elimination of sterols in the medium and food. Under sterol-free conditions, the first generation of worms developed from eggs to adults without external cholesterol. In the second generation they become dauer-like larvae but molting was incomplete. We found that replacing cholesterol with its natural metabolite lophenol, a methylated sterol, induced all worms to form regular dauer larvae. Using the effect of lophenol on growth, we could partially purify activity supporting the reproduction and determine the role of sterols during dauer larva formation and longevity. In the absence of this hormone, the nuclear hormone receptor DAF-12 is derepressed and thereby activates the dauer formation program. Active DAF-12 triggers in neurons the nuclear import of DAF-16 that contributes to dauer differentiation. Thus, the effect of lophenol allowed us to reveal a novel function of DAF-16 downstream of DAF-12 that is required for the execution of the dauer program but has no effect on life span.

## Results

### Worms Grown without Cholesterol for Two Generations Become Dauer-Like Larvae with Incomplete Molting

**Figure 1:**
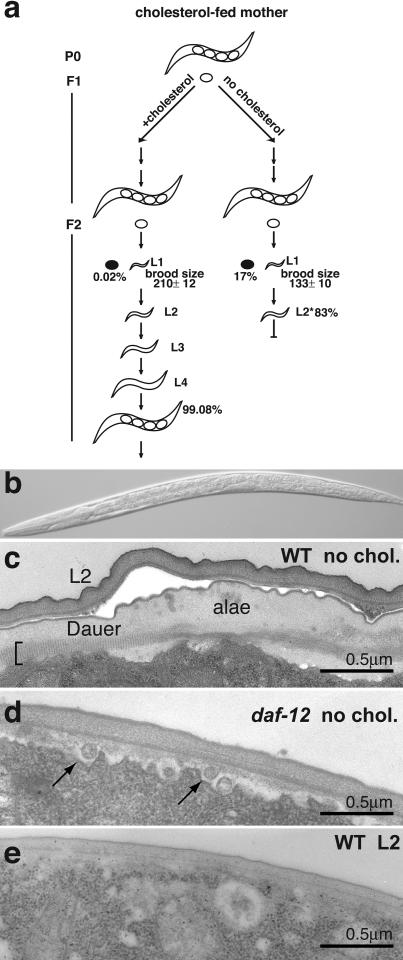
Depletion of Cholesterol Leads to Formation of Dauer-Like Larvae (A) For worms grown on plates without cholesterol, the first-generation worms (F1) laid fewer eggs than normal (133 ± 10 versus 210 ± 12) and more eggs failed to hatch. Filled ovals depict unhatched eggs; 17% of eggs laid by cholesterol-depleted worms failed to hatch in comparison to 0.02% of those laid by cholesterol-fed worms. The second generation of worms (F2) arrested after the completion of the L1-to-L2 molt. (B) Light micrograph of an arrested L2 larva. (C) Electron micrograph of the lateral cuticle of an arrested L2 larva 5 d after arrest, showing two cuticles. The outer cuticle resembles that of an L2, which has no alae, and the inner cuticle resembles the dauer cuticle with its distinctive striated layer (bracket) and an incomplete dauer ala. (D) Electron micrograph of an arrested L2 *daf-12* mutant grown without cholesterol. Arrows indicate vesicles beneath the cuticle which are not present in normal larvae. (E) Electron micrograph of a wild-type L2 larva grown with normal cholesterol.

In order to establish sterol-free growth conditions, we extracted traces of sterols from agarose and used defined medium for propagation of Escherichia coli to be fed to worms (see Materials and Methods). When eggs derived from mothers grown on normal plates (5 μg/ml of cholesterol; approximately 13 μM) hatched at low population densities on sterol-free plates seeded with bacteria, the first generation of worms developed from eggs to adults without external cholesterol. These adults, however, laid only about 60% as many eggs as normal, and 17% of the total eggs laid did not hatch (Figure 1A). The second-generation larvae completed their L1-to-L2 molt but then all arrested their development (Figure 1A, no cholesterol). Previous studies showing a weaker effect of cholesterol depletion in the second generation (Yochem et al. 1999; Crowder et al. 2001; Shim et al. 2002; Merris et al. 2003) might be due to contaminating sterols.

Figure 1B shows an F2 larva grown on a cholesterol-depleted plate. The arrested larvae are similar in size and appearance to L2 larvae grown on cholesterol. The number of cells in their gonads varied between five and 25 with an average of ten, similar to normally grown L2 larvae (Kimble and Hirsh 1979). These larvae stopped pharyngeal pumping after 3–5 d and became immobile after 7 d. If they were transferred to cholesterol-containing plates within the first 2–3 d, larvae reversed their arrest and matured to fertile adults. The reversal required as low as 20 nM cholesterol.

**Figure 3:**
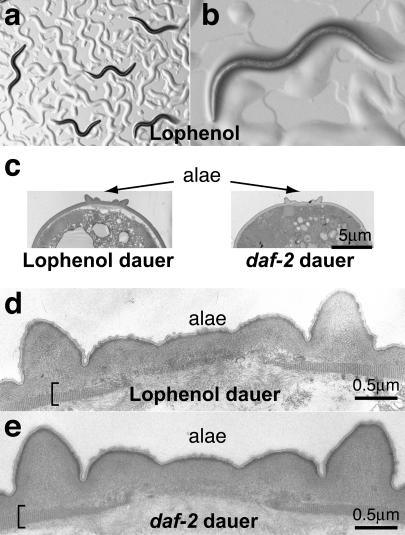
Wild-Type Worms Form Regular Dauer Larvae When Grown with Lophenol Replacing Cholesterol (A and B) Light microscopy of the second generation of worms grown on lophenol. Note low population density and ample bacteria on the plates (bacteria get swept into piles resembling worm tracks on agarose plates). (C–E) Electron micrographs of lophenol-grown and *daf-2* dauer larvae. The alae and the striated layer (bracket) are indistinguishable from those of regular dauer larvae, with extended outer projections.

The arrested larvae had a double cuticle (Figure 1C). The outer cuticle looks like a normal L2 cuticle and the inner cuticle has the characteristics of the normal dauer larva cuticle, including partially developed alae (for comparison, see Figure 3D) and the distinctive striated layer found only in dauer larvae (Cassada and Russell 1975) (Figure 1C). Other dauer-like features of these arrested larvae include constriction of the gut and unstained gut granules (unpublished data). Occasionally, animals were found with partially shed cuticles (<5%; unpublished data). In contrast to normal dauer larvae (Swanson and Riddle 1981), these arrested larvae were sensitive to treatment with 1% sodium dodecyl sulphate (SDS), perhaps because this shedding defect prevents complete dauer cuticle maturation.

We then asked whether the dauer features of the arrested larvae depend on *daf-12.* In the absence of cholesterol the second generation of *daf-12* worms arrested with only one cuticle similar to that of normal L2 (Figure 1D and 1E). These results show that on noncrowded plates with ample food, the absence of cholesterol causes L2 larvae to enter the normal dauer pathway utilising DAF-12.

In summary, our results imply that cholesterol, or cholesterol derivatives, are essential either for the development of reproductive adults or for the prevention of dauer larva formation. In addition, cholesterol derivatives are needed to shed the L2 cuticle.

### Cholesterol Depletion Leads to Reduced Levels of Nonmethylated Sterols and Accumulation of Methylated Sterols

**Figure 2:**
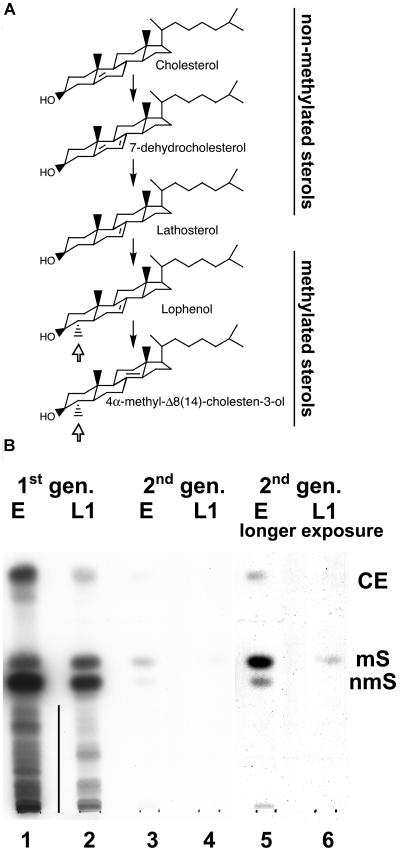
Depletion of Cholesterol Is Associated with a Decrease of Nonmethylated Sterols (A) Nematode-specific biosynthesis of 4-methylated sterols from exogenously added cholesterol. Open arrow shows the methylation at the fourth position. A vertical line indicates hydrophilic metabolites of cholesterol. (B) Cholesterol metabolism in the first (lanes 1 and 2) and the second (lanes 3–6) generations of worms derived from mothers fed with radioactive cholesterol. CE, cholesteryl esters; mS, methylated sterols (lophenol, 4-methylcholestenol); nmS, nonmethylated sterols (cholesterol, 7-dehydrocholesterol, lathosterol). The position of these compounds on TLC was determined by chromatography of cholesteryl stearate, lophenol, and cholesterol. E, eggs; L1, L1 larvae.

We investigated the metabolism of cholesterol in worms under conditions of cholesterol depletion. Eggs derived from mothers fed with radioactive cholesterol were put on cholesterol-depleted plates, where they grew for another generation to finally produce arrested L2/dauer larvae. The metabolism of sterols was followed by thin-layer chromatography (TLC). It has been previously established that C. elegans metabolises exogenously added cholesterol by methylating the A-ring at the fourth position and rearranging the double bond to form lophenol as the major product (Figure 2A) (Chitwood et al. 1983). In eggs and L1 larvae of the first generation (Figure 2B, lanes 1 and 2, respectively) or under conditions where cholesterol is present in the food permanently (unpublished data), methylated sterols (ms; lophenol and 4α-methyl-Δ8,14-cholestenol) are found in much lower amounts than nonmethylated sterols (nms; cholesterol, 7-dehydrocholesterol, and lathosterol). Quantification of radiographs revealed that under these conditions ms represent only 1%–3% of the total radioactivity. Cholesterol is also metabolised to a number of more hydrophilic derivatives (indicated by a vertical line), the identities of which are still unknown. In eggs derived from the second generation we observed two major changes: (i) The total radioactivity decreased and (ii) the fraction of nms showed a stronger decrease than that of ms (Figure 2B). The proportion between ms and nms changed even more dramatically in the L1 larvae of the second generation (Figure 2B, lanes 3–6). Here, about 95% of radioactivity was found as ms (lane 6). Note that the total radioactivity is on the limit of detection. Thus, upon cholesterol depletion the relationship between amounts of nms versus ms is altered.

### Substitution of Cholesterol by a Methylated Sterol, Lophenol, Leads to Dauer Larva Formation in the Second Generation

To test how nms influence dauer larva formation, we grew worms on plates with all cholesterol replaced by the methylated sterol lophenol (Figure 2A). When eggs from normally grown hermaphrodites were placed on lophenol plates, the first generation of worms was indistinguishable from that grown on cholesterol. They had normal brood size and normal morphology. In the second generation, however, the entire population completed two molts and became dauer larvae despite sufficient food and low population density (Figure 3A and 3B). Even on plates with a single worm, the individual developed into a dauer larva. These dauer larvae formed on lophenol plates had the distinct skinny shape, their pharynx was constricted, and they had very rare pharyngeal contractions, as detected earlier for dauer larvae formed by starvation (Keane and Avery 2003). Electron microscopy showed that they had the characteristic alae (Figure 3C) and striations of the normal dauer cuticle (compare Figure 3D and 3E). They were also resistant to SDS treatment like normal dauer larvae. These results show that, like cholesterol starvation, growth on lophenol leads to dauer larva formation. Unlike cholesterol starvation, however, lophenol allows shedding of the L2 cuticle to form normal dauer larvae.

The formation of dauer larvae by growth on 13 μM lophenol was prevented completely by adding cholesterol or its immediate precursor, lathosterol (see Figure 2A), in amounts as low as 20 nM. Under these conditions all the worms matured to fertile adults. The presence of contaminating nms in plates could be the reason why others did not find dauer larvae when worms were grown on lophenol (Merris et al. 2003).

The dauer larvae formed on lophenol plates resumed normal growth when transferred to cholesterol plates, although it required 3–4 d for them to reinitiate development, in contrast to the typical 15 h for normal dauer larvae.

### Methylation of the Fourth Position of Sterols Is Not Obligatory for Dauer Larva Formation

**Figure 4:**
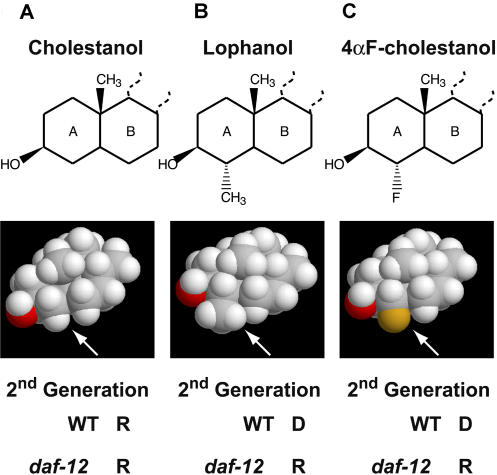
Methylation of the Fourth Position of Cholestanol Is Not Required for Dauer Larva Formation Structural formulae and space-filling models of (A) cholestanol, (B) lophanol, and (C) 4αF-cholestanol. Abilities to support reproductive growth or dauer formation in the second generation are indicated. R, reproduction; D, dauer larva.

The observation that small amounts of nms can prevent dauer larva formation on lophenol suggests that the lack of a cholesterol derivative which cannot be produced from lophenol is causing dauer larva formation. However, it is possible that lophenol itself actively induces dauer larva formation and methylation of sterols in the 4α position is necessary for this process. In order to distinguish between these alternatives we synthesised 5α-cholestan-3β-ols with a methyl group or fluorine substituted in 4α position (Figure 4) and fed them to worms. We decided to use saturated sterols because they are much more easily accessible for chemical synthesis (for details of synthesis, see Protocol S1). Cholestanol (Figure 4A) and lophanol (Figure 4B) have similar effects on growth as their homologues cholesterol and lophenol, respectively. The former supports reproductive growth, whereas the latter induces dauer formation. Remarkably, when fed with 4αF-cholestanol (Figure 4C), worms in the second generation produced dauer larvae. Fluorinated compounds, except in very rare cases, are not susceptible to chemical modifications by living organisms and therefore 4αF-cholestanol cannot be methylated. The fluorine atom is less bulky than the methyl group (Figure 4, space-filling models) and differs from the latter in its chemical properties. Thus, it is not the methylation of a sterol in the fourth position per se that is required for the formation of a dauer larva, but rather its accessibility is necessary to prevent this process. We suggest that cholesterol is normally metabolised in two distinct pathways: a pathway forming lophenol, and a pathway forming a steroid hormone. This hormone is required for maintaining reproductive growth and cannot be produced from ms.

### Partial Purification of Gamravali, an Activity That Promotes Reproduction

**Figure 5:**
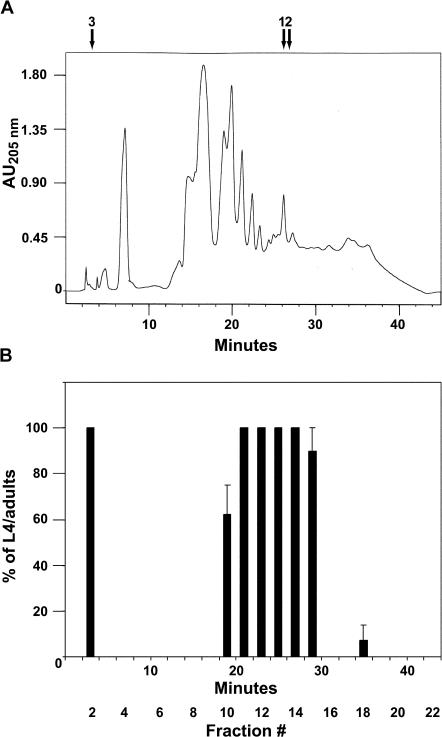
Partial Purification of Gamravali (A) Lipidic extract of worms was separated by HPLC using a C_18_ reverse-phase column. Retention times of (1) 7-dehydrocholesterol, (2) cholesterol/lathosterol, and (3) ecdysone/estradiol/testosterone are indicated with arrows. (B) Fractions of 2 min from the chromatography were assayed for the activity to rescue the formation of dauer larvae induced in the presence of lophenol.

The effect of lophenol on growth gave us a unique opportunity to purify the hormone (activity) required for reproductive growth. The rationale of our approach was to rescue the formation of dauer larvae induced in the presence of lophenol by a substance derived from a lipidic extract of worms. Obviously, this substance should differ from cholesterol and its direct metabolites such as 7-dehydrocholesterol and lathosterol. The lipidic extract of worms (see Materials and Methods) was fractionated using high-performance liquid chromatography (HPLC) on a reverse-phase C_18_ column (Figure 5A), and fractions mixed with lophenol were fed to L1 larvae of the second generation that were grown on lophenol. As seen in Figure 5B, two major peaks of activity rescuing dauer larva formation were detected. The major peak, according to retention times (23–30 min), should contain cholesterol, lathosterol, and 7-dehydrocholesterol. Another peak at the beginning of the gradient, however, is much more hydrophilic than major metabolic sterols. Two observations argue that this fraction is not contaminated by dietary cholesterol: (i) This region of the gradient never displayed activity even if the column was overloaded with cholesterol, and (ii) in contrast to cholesterol, active fraction #2 did not support reproductive growth alone, and instead many worms engulfed by the old cuticle were observed. This may be because another cholesterol-derived substance responsible for molting was missing.

We name this activity gamravali (from gamravleba, which means “reproduction” in Georgian; gamravali means “something supporting the reproduction”) because it is required for reproduction in worms. Currently we are attempting to determine the molecular formula of gamravali using mass spectroscopy. This task, however, is very demanding because of the tiny amounts of the substance in worms. Even more demanding will be the final identification of the structure by nuclear magnetic resonance or X-ray analysis. We estimated that the latter might require scaling of the preparation (see Materials and Methods) up to more than two orders of magnitude.

Our data indicate that gamravali is much more hydrophilic than sterols. Remarkably, retention times on the column of many mammalian steroids tested (pregnenolone, β-estradiol, testosterone, etc.) and the insect molting hormone ecdysone are very similar (Figure 5A). Thus, gamravali could be a polyhydroxylated sterol such as ecdysone, lack the hydrophobic side chain as in mammalian steroid hormones, or even contain a charged group. However, none of the compounds mentioned above or other commercially available steroids could rescue dauer formation in the presence of lophenol (see a list of tested compounds in Materials and Methods).

### A Mutant of *daf-12* Can Grow and Reproduce Normally on Lophenol for Many Generations, Whereas Several Daf-d Mutants Produce Dauer Larvae

The effect of lophenol on growth also made it possible to identify steps of the dauer formation pathway at which gamravali is required. For this we examined the phenotype of several dauer formation-defective (Daf-d) mutants when grown on lophenol. We assumed that mutants that are defective in metabolism of gamravali and thus act upstream of the hormone receptor should produce dauer larvae on lophenol. Mutants in genes acting downstream of the gamravali action should reproduce normally.

We first investigated the growth of a *daf-12* null mutant with lophenol as the sole source of sterols. DAF-12 as a putative nuclear hormone receptor is a good candidate to be a receptor for gamravali. In contrast to wild-type worms, mutants of *daf-12* grown on lophenol produced no dauer larvae and developed normally for more than seven generations. *daf-12* could also reproduce normally on lophanol and 4αF-cholestanol (see Figure 4). Therefore, *daf-12* acts downstream of gamravali depletion to promote dauer formation, leaving open the possibility that gamravali could be a ligand that inhibits the DAF-12. Our data also show that lophenol can substitute for all cholesterol functions except for the promotion of reproductive development.

In contrast to *daf-12,* other Daf-d mutants, such as *daf-22, daf-6, daf-10, daf-3,* and *daf-5* developed into dauer larvae when grown on lophenol. According to genetic studies all these genes are upstream of *daf-12* in the pathway. *daf-22* and *daf-6* cannot produce or sense the dauer-inducing pheromone, respectively (Golden and Riddle 1985; Perkins et al. 1986). DAF-3 and DAF-5 are SMAD transcription factor and its regulator Ski, which antagonise TGF-β action (Patterson et al. 1997; Da Graca et al. 2004). The functions of these genes could be to inhibit gamravali production when the dauer pathway is initiated by starvation or overcrowding. Growth on lophenol alone would mimic this situation and result in dauer formation since gamravali cannot be made from lophenol.

### Mutant *daf-16* Produces Defective Dauer Larvae on Lophenol and the Latter Induces Entry of DAF-16 into Nuclei of Neurons in a DAF-12–Dependent Manner

**Figure 6:**
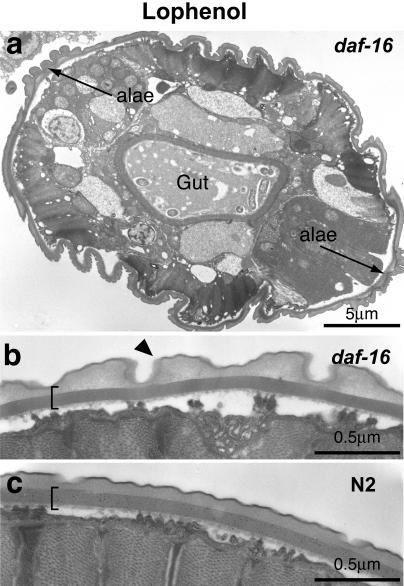
Mutant *daf-16* Worms Grown on Lophenol Form Defective Dauer Larvae (A) Low-magnification electron micrograph of lophenol-grown *daf-16*. The alae are defective although the striated layer (bracket) is visible. Note that the gut is not constricted and contains remnants of food. (B and C) High-magnification electron micrographs of lophenol-grown *daf-16* and wild-type dauer larvae. Arrowhead indicates an annular structure.

Somewhat different results were obtained with null mutants of *daf-16* grown on lophenol. In the second generation, neither reproductive adults nor regular dauers were observed. The larvae were fully susceptible to the SDS treatment and their morphology displayed several abnormalities in comparison to regular dauer larvae (Figure 6). They did not have alae of normal morphology (compare Figure 6A and 3E), although a striated layer characteristic of the dauer state was visible. The gut was not constricted as in regular dauer larvae (Figure 6A). Remarkably, the cuticle displayed annular structures (Figure 6B, arrowhead) characteristic of adults but never detected in dauer larvae (compare Figure 6B and 6C). One in about 400 worms would occasionally mature and produce a few eggs that never hatched. Thus, in the absence of DAF-16 only a partial, defective dauer larva can be produced on lophenol. Similar defective dauers (although with very low efficiency) were produced by pheromone-treated *daf-16* (Vowels and Thomas 1992). Our data indicate that in the absence of cholesterol or its derivatives other than lophenol, *daf-16* is still needed for normal differentiation of dauer larvae. Thus, DAF-16 should have activity downstream of the sterol requirement.

Genetic epistasis analysis suggested that *daf-16* functions upstream of *daf-9,* which in turn acts upstream of *daf-12* (Gerisch et al. 2001; Jia et al. 2002). Moreover, it has been proposed that DAF-16 inhibits DAF-9, a cytochrome P450 that could be involved in the synthesis and/or degradation of a gamravali-like ligand for DAF-12. Consequently, gamravali should be required downstream of DAF-16 function. However, our data (Figure 6) imply that, in addition to the regulation of sterol biosynthesis, DAF-16 acts downstream of DAF-12 and is involved in the differentiation of dauer larvae.

**Figure 7:**
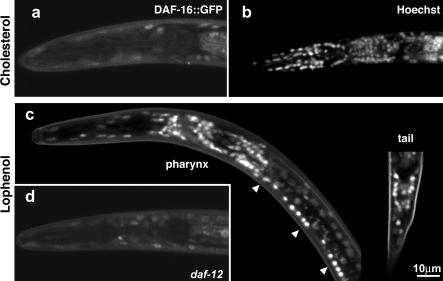
Growth on Lophenol Induces the Accumulation of DAF-16 in the Nuclei of Neurons in a DAF-12–Dependent Manner (A) When grown on cholesterol, the transgenic line DAF-16a::GFP/b^KO^ displays a diffuse staining in the cytoplasm and nuclei of many cells (only the pharynx region of an L3 larva is shown). (B) Staining of a larva of similar age by Hoechst. Note many nuclei in the pharynx. (C) The DAF-16a::GFP/b^KO^ line grown on lophenol shows strong staining of nuclei in neurons of the pharynx, tail, and ventral cord of a dauer larva. (D) An L3 larva of DAF-16a::GFP/b^KO^ in a *daf-12* null background grown on lophenol. Note the diffuse fluorescence in the pharynx cell similar to that shown in (A).

Under reproductive conditions, DAF-16 is found in both the cytoplasm and the nucleus, whereas upon activation by the IGF-1 or the TGF-β pathways the protein is accumulated in the nucleus (Henderson and Johnson 2001; Lee et al. 2001; Lin et al. 2001). We asked whether the growth on lophenol had a similar effect on the cellular distribution of DAF-16. In order to answer this question, we made use of a transgenic line expressing a DAF-16::GFP fusion protein (Lin et al. 2001). In L3 larvae grown on cholesterol, DAF-16::GFP showed diffuse fluorescence throughout many cells, as reported previously (Figure 7A). In contrast, in the second generation of worms grown on lophenol, DAF-16::GFP is localised in nuclei of neurons of the pharynx, ventral cord, and tail (Figure 7C). Lophenol had a very weak effect on the accumulation of DAF-16 in the nuclei of other cells (e.g., gut or muscles).

Is the nuclear accumulation of DAF-16 upon growth on lophenol dependent on the activation of DAF-12? DAF-16::GFP showed diffuse staining in a *daf-12* null mutant grown on lophenol (compare Figure 7D and 7A). Our data therefore indicate that the activation of DAF-12 induced by the absence of gamravali leads to accumulation of DAF-16 in the nuclei of neurons.

Since the double *daf-16;daf-12* mutant grown on lophenol did not produce dauer larvae and could grow on this sterol for many generations, the phenotype of *daf-16* observed in the absence of hormone (see Figure 6A and 6B) depends on the activity of *daf-12.* These results imply that the dauer formation process is initiated by DAF-12 but needs nuclear import of DAF-16, which in turn contributes to dauer differentiation, presumably through transcriptional regulation in the nucleus.

### Growth on Lophenol Does Not Extend the Life Span of Worms

*daf-2* mutants have a life span that is approximately twice as long as that of the wild-type worms (Kenyon et al. 1993), and in addition mutants display strong intrinsic thermotolerance (Gems et al. 1998). This effect is attributed to the activation of DAF-16 in a *daf-2* mutant, since a double *daf-16;daf-2* mutant suppresses this phenotype. Does the nuclear accumulation of DAF-16 in neurons when grown on lophenol have a similar effect on life span and thermotolerance? In wild-type worms of the first generation grown on cholesterol or lophenol we could not detect significant differences in the mean life span (21.0 ± 1.8 d and 20.3 ± 1.6 d for cholesterol and lophenol, respectively). It must be noted, however, that worms grown in the absence of cholesterol and the presence of lophenol do not have a developmental phenotype in the first generation and therefore may have some maternal rescue of adult life span. Because the second generation does not grow to adulthood (forms dauer larvae), the definitive experiment cannot be performed. Growth on lophenol also had no influence on the intrinsic thermotolerance of worms at 39 °C. Thus, the activation of DAF-16 induced by the absence of gamravali might have different physiological consequences than its activation by diminished IGF-1 signalling.

## Discussion

### Worms Need Tiny Amounts of Sterols for Survival

In our attempts to understand the role of cholesterol in nematodes we developed strict sterol-free conditions for growth by combination of the extraction of agarose with organic solvents and the growing of bacteria on defined media. Under these conditions, the first generation of worms grew relatively normally and only the second generation arrested as dauer-like larvae. Thus, the amount of sterols deployed by mothers into embryos is sufficient not only for the survival of the first generation but even for the embryonic development of about 130 embryos that reach the L2 stage in the second generation. This makes cholesterol unlikely to be an indispensable structural component in most worm membranes, although it could play a structural role in cell types where it is concentrated (Matyash et al. 2001). These results are difficult to reconcile with the widespread role of cholesterol and other nms as essential structural components of the plasma membrane. Presumably, C. elegans can regulate membrane properties in response to temperature changes by altering fatty acid composition of phospholipids (Tanaka et al. 1996). Future investigations should clarify what components of nematode membranes substitute for structural functions of cholesterol and whether mechanisms exploited by nematodes to control membrane properties are also serving an analogous purpose in higher eukaryotes.

### Hormonal Regulation of Dauer Larva Formation: Gamravali versus Lophenol

**Figure 8:**
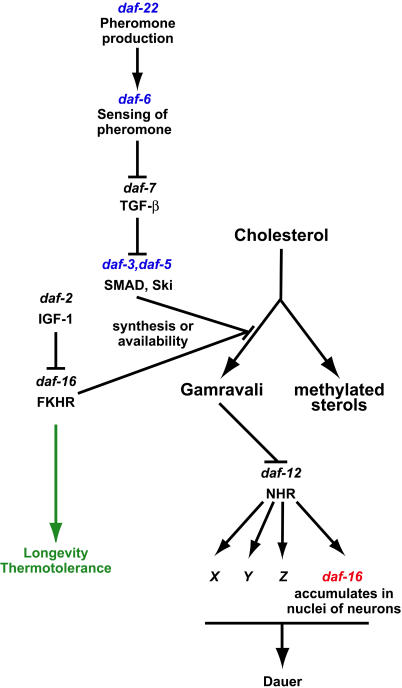
Cross Talk between Two Signalling Pathways in the Process of Dauer Larva Formation Pheromone accumulated under the conditions of overcrowding or starvation induces the inhibition of gamravali production via the TGF-β pathway. Genes, mutants of which produced dauer larvae on lophenol, are shown in blue. Activated by the absence of gamravali, DAF-12 initiates the process of dauer larva production. One of its activities is to recruit DAF-16 into nuclei of neurons (shown in red). The insulin-like pathway has several physiological functions, among them the regulation of longevity and thermotolerance, and could be involved in the process of dauer formation by regulating the levels of gamravali via DAF-16.

Our results obtained by growing wild-type and mutant worms without cholesterol, with cholesterol replaced by lophenol, and with lophenol supplemented by gamravali demonstrate unequivocally that the decision to enter diapause is regulated by a sterol-derived hormone(s). We propose the following model to explain these results (Figure 8). Gamravali derived from cholesterol acts to promote reproduction and prevent dauer larva formation by inhibiting the nuclear hormone receptor DAF-12. The effect of external signals that induce dauer formation, starvation, and overcrowding is to prevent gamravali production, thus preventing reproduction and promoting entry into diapause. According to this model, growth on lophenol resembles the absence of gamravali. Although it is formally possible that lophenol or ms derived from it could induce dauer formation, this is unlikely for three reasons: (i) Dauer larvae are not induced in the first generation on lophenol, (ii) cholesterol and gamravali at concentrations less than 1/600 that of lophenol prevent dauer larva formation, and (iii) the 4α-fluoro derivative can substitute for lophenol. It is much more likely that lophenol supports all the functions of cholesterol, structural and hormonal, except promoting reproductive growth, since *daf-12* null mutants grow and reproduce normally on lophenol. The *daf-9* gene, which encodes a cytochrome P450 (Gerisch et al. 2001; Jia et al. 2002), could be involved in the production of gamravali. Remarkably, expression of DAF-9 in *daf-7* and *daf-2* could rescue their Daf-c phenotype (Gerisch and Antebi 2004; Mak and Ruvkun 2004). We did not detect any gross-level changes of cholesterol metabolism in the double null *daf-9 daf-12* strain (Figure S1). However, since DAF-9 is expressed predominantly in only a small subset of cells (Gerisch et al. 2001; Jia et al. 2002), differences in overall cholesterol metabolism might be small and require more sensitive assays.

### The Place and Role of *daf-16* in the Dauer Formation Pathway

Our data uncover a dual role for DAF-16, first during the reproductive/dauer decision and second during dauer differentiation. It has been established that DAF-16 acts via the insulin-dependent pathway and is involved in the inhibition of hormone production (Gerisch et al. 2001; Jia et al. 2002), thereby controlling the reproductive/dauer decision (Figure 8, right branch). Our results show that, in addition, DAF-16 functions downstream of DAF-12 so that activation of the latter recruits it into neuronal nuclei (Figure 8, shown in red). The process of dauer formation, thus, is initiated by DAF-12 but needs DAF-16.

A direct physical interaction between nuclear hormone receptors and forkhead domain (FOXO) transcription factors has recently been reported (Schuur et al. 2001; Zhao et al. 2001; Dowell et al. 2003; Li et al. 2003). It is tempting to speculate that DAF-12 and DAF-16 can interact physically and that the activated DAF-12 can retain DAF-16 in the nucleus. Consistent with this, DAF-12 and DAF-16 have been coimmunoprecipitated in a recent in vitro study (Dowell et al. 2003).

### Sterols and Longevity

According to a current view, *daf-16* is a major regulator of the longevity process (Lin et al. 1997; Ogg et al. 1997). Reduction of DAF-2/IGF-1 signalling leads to activation of DAF-16 and to near-doubling of the life span of worms (Kenyon et al. 1993; Morris et al. 1996). The inhibition of insulin receptor activity leads to the redistribution of FOXO transcription factors from cytoplasm into the nucleus and thus is a prerequisite for their activity (Henderson and Johnson 2001; Lee et al. 2001; Lin et al. 2001). Our data show that in worms grown on lophenol, DAF-16 accumulates strongly in neuronal nuclei. The growth on lophenol, however, has no consequences on the length of the life span. A plausible explanation for this observation is that the activity of *daf-16* influencing life span is tissue specific. In a recent study, Libina et al. (2003) have expressed DAF-16 in a *daf-16;daf-2* double mutant under different tissue-specific promoters. Whereas expression of DAF-16 in the intestine led to the extension of the life span, expression in the neurons had no effect on longevity. This is consistent with our data showing that the DAF-12–dependent nuclear import of DAF-16 in neurons activates a different program from that in the intestine.

## Materials and Methods

### 

#### Materials

Lophenol was purchased from Research Plus (Manasquan, New Jersey, United States). Electrophoresis-grade ultraPURE agarose was the product of Life Technologies (Paisley, Scotland, United Kingdom). Dulbecco's medium (DMEM) was from Invitrogen (Karlsruhe, Germany). Sterols, steroids, and antioxidant BTH were from Sigma (Sigma-Aldrich Chemie, Taufkirchen, Germany).

All mutants except *daf-12 (rh61rh411)* and *daf-9 (dh6) daf-12 (rh61rh411)* were obtained from the *Caenorhabditis* Genetics Center. *daf-12* and the double mutant *daf-9 daf-12* were a kind gift of Adam Antebi (Max Planck Institute for Molecular Genetics, Berlin, Germany). The following mutant strains were used throughout the study: *daf-22 (m130)*II, *daf-6 (e1377)*X, *daf-3 (mgDf90)*X, *daf-5 (e1386)*II, *daf-10 (e1387)*IV, *daf-12 (rh61rh411)*X, *daf-2 (e1370ts)*III, and *daf-16 (mgDf50)*I. For imaging studies strains *daf-16 (mu86)*I; *muIs71*[*pKL99*(*daf-16a::GFP/bKO*)+*pRF4(rol-6)*]X and *daf-16 (mu86)*I; *muIs61 (daf-16::GFP (pKL78)+rol-6(pRF4)* were tested. Since the former, *daf-16a::GFP/bKO,* gave a brighter signal, as previously reported (Henderson and Johnson 2001; Lee et al. 2001; Lin et al. 2001), results obtained with this strain are presented throughout the study.

#### Preparation of sterol-depleted and sterol-containing plates for the propagation of worms

Wild-type N2 Bristol and mutant strains were routinely propagated on NGM-agar plates as described in Brenner (1974). To obtain cholesterol-free conditions, agar was replaced by agarose (extracted three times with chloroform) and peptone was omitted from plates. An overnight culture of the NA22 strain of E. coli was grown on a sterol-free culture medium DMEM. Bacteria were rinsed with M9 medium before use.

For preparation of sterol-containing plates, cholesterol or lophenol was dissolved in methanol to the concentration of 5 mM and mixed 1:1 (v/v) with cholesterol-free bacterial suspension in M9. After evaporation of methanol in SpeedVac, the suspension was mixed with fresh bacteria to obtain the desired end concentration of tested substance. Bacterial suspensions were spread on cholesterol-free agarose plates.

#### Light, fluorescence, and electron microscopy

Light and confocal fluorescence microscopy were done using Zeiss Axioplan and Axiovert LSM 510 microscopes, respectively. For nuclear labelling, larvae were grown on medium containing 5 μg/ml of Hoechst (Molecular Probes, Eugene, Oregon, United States). One hour before imaging, larvae were transferred to medium without Hoechst, washed briefly with M9 medium, anaesthetised with 40 mM sodium azide in M9, and mounted on agarose pads. For electron-microscopic studies arrested larvae were washed two times with M9, harvested by centrifugation, and mixed with an equal volume of 2×Fixative (5% glutaraldehyde, 2% paraformaldehyde in M9). Worms were cut at room temperature with a razor blade on a microscopic slide, transferred to a centrifuge tube, and incubated for 2 h in a refrigerator. Afterwards worms were centrifuged and embedded in EmBed-812 (EMS, Ft. Washington, Pennsylvania, United States). Images were acquired by Tecnai 12 (FEI, Eindhoven, The Netherlands) or Phillips 400 electron microscopes.

#### TLC of cholesterol metabolites from *C. elegans*

To investigate cholesterol metabolism in *C. elegans,* 10-cm NGM agar plates were prepared without cholesterol. A quantity of 300 μl of bacterial suspension containing 13 μM cholesterol was supplemented with 4 μCi of [^3^H]-cholesterol (70 Ci/mmol; Amersham Biosciences Europe, Freiburg, Germany). Worms were harvested from the plates with M9 medium and subjected to three cycles of freezing-thawing, and lipids were extracted by the Bligh and Dyer method (Bligh and Dyer 1957). Eggs derived from mothers fed with radioactivity were put on sterol-free plates and propagated for two generations. Equal amounts of eggs and L1 larvae estimated by counting of aliquots were extracted and analysed by TLC as described above.

TLC was performed on glass-backed plates of silica gel 60 (Merk, Darmstadt, Germany). Solvents used for the separation of cholesterol metabolites were chloroform-methanol (24:1).

After chromatography, plates were sprayed with a scintillator (Lumasafe, Lumac LSC B.V., Groningen, The Netherlands) and exposed to a film (Hyperfilm MP, Amersham Biosciences Europe, Freiburg, Germany).

We quantified relative amounts of ms and nms by scanning films exposed to radioactivity for short time and using Adobe Photoshop software.

#### Regio- and stereospecific synthesis of 4α-substituted 5α-cholestan-3β-ols

The synthesis of 4α-substituted 5α-cholestan-3β-ols is described in the Supporting Information.

#### Preparation and HPLC fractionation of a lipidic extract from worms

Worms of mixed population from 150 15-cm plates were collected by rinsing with ice-cold water and left overnight at 4 °C to sediment. The final volume of the sediment was about 150 ml. After decantation, aliquots of the worm suspension were transferred into 50-ml Falcon tubes and subjected to three cycles of freezing in liquid N_2_ and thawing by sonication in an ultrasound bath at 37 °C. Worm suspension was then transferred into a glass bottle, 19 volumes of methanol containing 10 μg/ml of antioxidant BHT was added, and extraction was performed overnight at room temperature under continuous agitation. Extract was separated from worm remnants by filtration through a Whatman GF/A glass filter and remnants were reextracted with a fresh portion of methanol. Methanol extracts were combined and extracted two times with one volume of hexane. The obtained hexane extract was washed twice with a methanol-water mixture (9:1), dried under N_2_ flow, and dissolved in 7 ml of hexane.

In order to dispose very hydrophobic substances, the extract was subjected to a solid-phase separation. A quantity of 200 μl of the hexane fraction was applied to a 20-ml LC-18-SPE cartridge (SUPELCO, Bellefonte, Pennsylvania, United States) equilibrated with methanol. Twenty millilitres of flow-through methanol was collected, dried under N_2_ flow, and dissolved in 200 μl of methanol. Two preps (400 μl) were subjected to reverse-phase HPLC chromatography on an Alliance 2695 solvent module (Waters GmbH, Eschborn, Germany) linked to a Waters 996 photodiode array detector using an XTerra Prep MS C_18_ 10 μm 10 × 250-mm column (Waters). The elution protocol was as follows: 15% solvent A (20% methanol in water) and 85% solvent B (methanol) for 11 min, a gradient from 85% B to 100% B in 11 min, and 100% B for 18 min. The flow rate was 5 ml/min. Fractions of 2 min were collected, dried, dissolved in 400 μl of isopropanol, and stored at −80 °C until use.

#### Assay for gamravali

Testing of the biological activity of HPLC fractions was performed in 12-well cell culture plates (Nunc, Roskilde, Denmark). Each well contained 1 ml of sterol-free agarose mixed with 0.1% tergitol. A quantity of 100 μl of HPLC fractions was added per well and dried in the laminar flow cabinet. Before seeding worms, 30 μl of sterol-free bacteria containing 10 μM lophenol was added to plates and left to stay overnight at room temperature.

Worms for the bioassay were prepared as follows. The first generation of adult worms grown on lophenol (see above) was bleached, and eggs were placed on sterol-free plates without food and were kept for 3 d to obtain synchronised L1 larvae.

About ten starved L1 larvae were placed in each well with HPLC fractions at room temperature. After 4 d worms were scored and the activity of fractions was represented as the percentage of worms that reached L4 or adult stages. Quadruplicates of each fraction per experiment were analysed.

#### Compounds tested to rescue the dauer larva formation in the presence of lophenol

Pregnenolone, testosterone, estrone, β-estradiol, progesterone, androstenol, vitamin D_3_, ecdysone, 20-hydroxyecdysone, 7α-hydroxycholesterol, 7β-hydroxycholesterol, 19-hydroxycholesterol, 20-hydroxycholesterol, 22-hydroxycholesterol, 24-hydroxycholesterol, 26-hydroxycholesterol, cholic acid, dehydrocholic acid, deoxycholic acid, litocholic acid, taurodeoxycholic acid, and chenodeoxycholic acid were tested. 7α-hydroxycholesterol, 19-hydroxycholesterol, and 26-hydroxycholesterol were from Steraloids (Newport, Rhode Island, United States); all others were from Sigma.

#### Generating a double null mutant for *daf-12* and *daf-16* and a transgenic line expressing DAF-16::GFP in *daf-12* null background

The double mutant *daf-16(mu86)*I; *daf-12 (rh61rh411)*X was generated by crossing *daf-16 (mu86)*I; *muIs71*[*pKL99(daf-16a::GFP/bKO)*+*pRF4(rol-6)*]X hermaphrodites with *daf-12 (rh61rh411)* males. Progeny displaying no Roller phenotype and fluorescence and able to grow on lophenol were selected. The *daf-16(mu86)* mutation was identified by PCR. Consequently, the mutations were verified by sequencing.

The double mutant *daf-16(mu86)*I; *daf-1*2 *(rh61rh411)*X was then used to generate *daf-16 (mu86)*I; *daf-12 (rh61rh411) muIs71*[*pKL99(daf-16a::GFP/bKO)*+*pRF4(rol-6)*]X worms by backcrossing to the original *daf-16 (mu86)*I; *muIs71*[*pKL99(daf-16a::GFP/bKO)*+*pRF4(rol-6)*]X. The mutations were identified and verified in a way similar to that used for the double mutant.

#### Life span and thermotolerance

The life span and thermotolerance of worms were investigated according to the method of Gems et al. (1998). Studies with N2 animals were performed on plates containing cholesterol or lophenol at 20 °C. Day 0 corresponded to L4 stage. The life spans of about 300 worms per condition were investigated.

## Supporting Information

Figure S1 Comparison of Cholesterol Metabolism in Wild-Type, *daf-12,* and Double Mutant *daf-9 daf-12* Worms (3.8 MB PDF).

Protocol S1 Regio- and Stereospecific Synthesis of 4α-Substituted 5α-Cholestan-3β-ols (114 KB DOC).

## Abbreviations

Daf-d: dauer formation defective
HPLC: high-performance liquid chromatography
ms: methylated sterols
nms: nonmethylated sterols
SDS: sodium dodecyl sulphate
TLC: thin-layer chromatography
