# Supplementary figures and images for: Sterol-Derived Hormone(s) Controls Entry into Diapause in Caenorhabditis elegans by Consecutive Activation of DAF-12 and DAF-16
Source: PLoS Biol. 2004 Sep 21;2(10):e280. doi: 10.1371/journal.pbio.0020280 (PMC517820; doi:10.1371/journal.pbio.0020280)

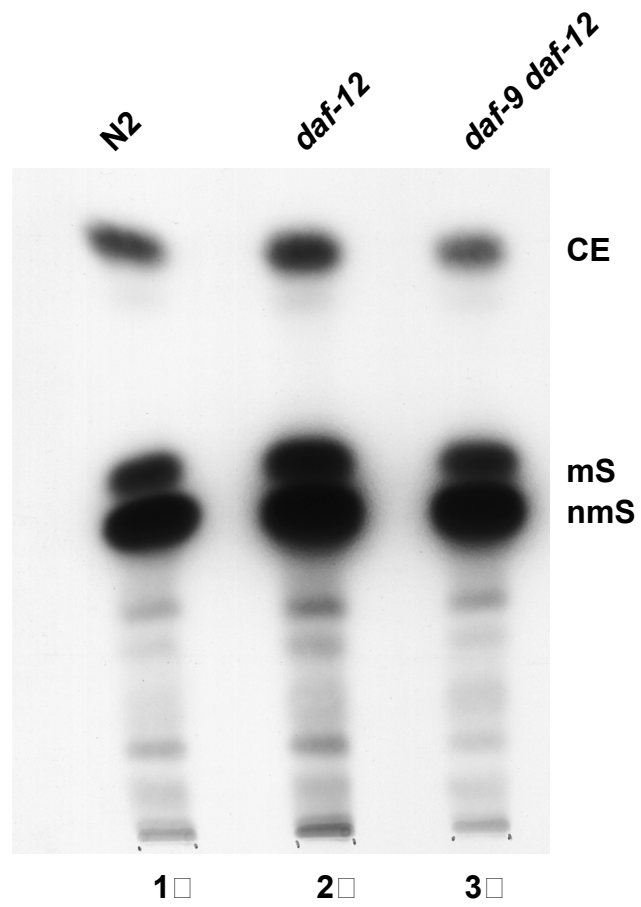

Supplement: Figure S1 — (3.8 MB PDF). [file pbio.0020280.sg001.pdf]
