# Supplementary material for: Sterol-Derived Hormone(s) Controls Entry into Diapause in Caenorhabditis elegans by Consecutive Activation of DAF-12 and DAF-16
Source: PLoS Biol. 2004 Sep 21;2(10):e280. doi: 10.1371/journal.pbio.0020280 (PMC517820; doi:10.1371/journal.pbio.0020280)
Supplement: Protocol S1 — (114 KB DOC). [file pbio.0020280.sd001.doc]

Supplementary material

**Regio- and Stereospecific Synthesis of 4α-Substituted 5α-Cholestan-3β-ols**

Herein, we describe the regio- and stereospecific synthesis of 4α-substituted 5α-cholestan-3β-ols. Reduction of 4-cholesten-3-one (**1**) with lithium in liquid ammonia and subsequent alkylation with iodomethane according to the procedure developed by Stork1-3 afforded 4α-methyl-5α-cholestan-3-one (lophanone) (**2**) (Scheme 1).4, 5 Stereoselective reduction of compound **2** using lithium aluminum hydride provided as the major product 4α-methyl-5α-cholestan-3β-ol (lophanol) (**3**),4 which has been previously isolated from diverse natural sources.5-11

Scheme 1

Lithium–ammonia reduction of 4-cholesten-3-one (**1**) followed by quenching with chlorotrimethylsilane^12^ led to the trimethylsilyl enol ether **4** (Scheme 2). Electrophilic fluorination of compound **4** using 1-fluoro-2,4,6-trimethylpyridinium triflate afforded 4α-fluoro-5α-cholestan-3-one (**5**).13 Stereoselective reduction of **5** provided 4α-fluoro-5α-cholestan-3β-ol (**6**).

Scheme 2

**4α-Methyl-5α-cholestan-3-one (2)**

Lithium (50 mg, 7.2 mmol) was added to a solution of 4-cholesten-3-one (**1**) (1.00 g, 2.60 mmol) in anhydrous THF (20 ml) and ammonia (25 ml) at –78°C. After warming to
–35°C and stirring for 4 h under nitrogen, the solution was cooled to –78°C and iodomethane (5.17 g, 2.27 ml, 36.4 mmol) was added. Ammonia was allowed to evaporate overnight, water was added, and the aqueous layer was acidified by addition of 10% HCl. The aqueous layer was extracted with ether three times and the combined organic layers were dried over sodium sulfate. Evaporation of the solvent and flash chromatography (pentane/ether, 10:1) of the residue on silica gel afforded 4α-methyl-5α-cholestan-3-one (**2**) as colorless crystals, yield: 402 mg (39%), mp: 103-105°C. IR (ATR): ν = 2931, 2866, 2848, 1709, 1467, 1441, 1383, 1376, 959 cm^–1^. ^1^H-NMR (500 MHz, CDCl_3_): δ = 0.66 (s, 3 H), 0.68 (m, 1 H), 0.848 (d, *J* = 6.6 Hz, 3 H), 0.853 (d, *J* = 6.6 Hz, 3 H), 0.89 (d, *J* = 6.5 Hz, 3 H), 0.93-1.01 (m, 2 H), 0.96 (d, *J* = 6.5 Hz, 3 H), 1.03-1.20 (m, 8 H), 1.05 (s, 3 H), 1.22-1.27 (m, 1 H), 1.31-1.39 (m, 6 H), 1.48-1.59 (m, 4 H), 1.64-1.66 (m, 1 H), 1.71-1.75 (m 1 H), 1.77-1.84 (m, 1 H), 1.95-2.04 (m, 2 H), 2.27-2.32 (m, 2 H), 2.43 (m, 1 H). ^13^C-NMR and DEPT (125 MHz, CDCl_3_): δ = 11.50 (CH_3_), 12.06 (CH_3_), 12.70 (CH_3_), 18.65 (CH_3_), 21.38 (CH_2_), 22.56 (CH_3_), 22.82 (CH_3_), 23.82 (CH_2_), 24.19 (CH_2_), 25.65 (CH_2_), 28.01 (CH), 28.27 (CH_2_), 31.94 (CH_2_), 34.87 (CH), 35.78 (CH), 36.14 (CH_2_), 36.34 (C), 38.06 (CH_2_), 39.28 (CH_2_), 39.50 (CH_2_), 39.93 (CH_2_), 42.53 (C), 45.06 (CH), 53.58 (CH), 54.08 (CH), 56.24 (CH), 56.32 (CH), 213.80 (C=O). MS (80°C): *m/z* (%) = 400 (60) [M^+^], 385 (24), 260 (12), 246 (52), 245 (100), 231 (24). HRMS: *m/z* [M^+^] calcd for C_28_H_48_O: 400.3705; found: 400.3709. Analysis calcd for C_28_H_48_O: C 83.93, H 12.07; found: C 83.87, H 12.07.

**4α-Methyl-5α-cholestan-3β-ol (3)**

A 1M solution of lithium aluminum hydride (0.75 ml, 0.75 mmol) was added to a solution of 4α-methyl-5α-cholestan-3-one (**2**) (300 mg, 0.75 mmol) in anhydrous THF (10 ml) at 0°C. After stirring for 4 h at 0°C under an argon atmosphere, water and then 10% HCl were added. The aqueous layer was extracted with ether three times. The combined organic layers were washed with a saturated solution of sodium bicarbonate and dried over sodium sulfate. Removal of the solvent and purification of the residue by flash chromatography (pentane/ether, 5:1) on silica gel provided 4α-methyl-5α-cholestan-3β-ol (**3**) as colorless crystals, yield: 239 mg (80%), mp: 145°C. IR (ATR): ν = 3322, 2926, 2864, 2850, 1444, 1380, 1037, 1012, 957 cm^–1^. ^1^H-NMR (500 MHz, CDCl_3_): δ = 0.59 (m, 1 H), 0.63 (s, 3 H), 0.70 (m, 1 H), 0.79 (m, 1 H), 0.81 (s, 3 H), 0.847 (d, *J* = 6.6 Hz, 3 H), 0.852 (d, *J* = 6.6 Hz, 3 H), 0.88 (d, *J* = 6.5 Hz, 3 H), 0.91-1.15 (m, 10 H), 0.93 (d, *J* = 6.3 Hz, 3 H), 1.18-1.37 (m, 7 H), 1.43-1.57 (m, 4 H), 1.63-1.73 (m, 3 H), 1.77-1.82 (m, 2 H), 1.95 (dt, *J* = 12.6, 3.4 Hz, 1 H), 3.07 (ddd, *J* = 11.0, 10.0, 4.9 Hz, 1 H). ^13^C-NMR and DEPT (125 MHz, CDCl_3_): δ = 12.06 (CH_3_), 13.35 (CH_3_), 15.13 (CH_3_), 18.65 (CH_3_), 21.13 (CH_2_), 22.55 (CH_3_), 22.81 (CH_3_), 23.82 (CH_2_), 24.17 (CH_2_), 24.19 (CH_2_), 28.00 (CH), 28.28 (CH_2_), 31.06 (CH_2_), 32.23 (CH_2_), 34.84 (CH), 35.79 (CH), 35.99 (C), 36.16 (CH_2_), 36.82 (CH_2_), 39.22 (CH), 39.51 (CH_2_), 40.07 (CH_2_), 42.51 (C), 50.92 (CH), 54.55 (CH), 56.26 (CH), 56.53 (CH), 76.62 (CH). MS (150°C): *m/z* (%) = 402 (100) [M^+^], 400 (53), 387 (52), 385 (25), 369 (26), 262 (38), 248 (57), 247 (80), 246 (53), 245 (85), 231 (38), 229 (90). HRMS: *m/z* [M^+^] calcd for C_28_H_50_O: 402.3862; found: 402.3849. Analysis calcd for C_28_H_50_O: C 83.51, H 12.51; found: C 83.69, H 12.45.

**3-Trimethylsilyloxy-5α-cholest-3-ene (4)**

Lithium (40 mg, 5.7 mmol) was added to a solution of 4-cholesten-3-one (**1**) (1.00 g, 2.60 mmol) in anhydrous THF (20 ml) and ammonia (25 ml) at –78°C. The solution was warmed to –35°C, stirred for 2 h under a nitrogen atmosphere, and ammonia was evaporated by warming to r. t. After addition of THF (10 ml), the reaction mixture was cooled to –10°C, and triethylamine (1.05 g, 1.46 ml, 10.4 mmol) and chlorotrimethylsilane (1.13 g, 1.30 ml, 10.4 mmol) were added. The reaction mixture was stirred for 30 min, diluted by addition of ether (30 ml), washed with a saturated solution of sodium bicarbonate, and then with brine. The organic layer was dried over sodium sulfate and the solvent was evaporated. Flash chromatography (hexane with 1% triethylamine) of the residue on neutral alumina provided the trimethylsilyl enol ether (**4**) as colorless crystals, yield: 653 mg (55%), mp: 97-103°C. IR (ATR): ν = 2928, 2868, 1660, 1466, 1443, 1375, 1250, 1203, 923, 888, 841, 759 cm^–1^. ^1^H-NMR (300 MHz, benzene-d_6_): δ = 0.20 (s, 9 H), 0.59-0.68 (m, 1 H), 0.66 (s, 3 H), 0.83 (s, 3 H), 0.86-1.67 (m, 23 H), 0.91 (d, *J* = 6.6 Hz, 6 H), 0.99 (d, *J* = 6.5 Hz, 3 H), 1.77-1.87 (m, 1 H), 1.95-2.17 (m, 4 H), 4.68 (d, *J* = 1.0 Hz, 1 H). ^13^C-NMR and DEPT (75 MHz, benzene-d_6_): δ = 0.50 (3 CH_3_) 12.10 (CH_3_), 12.44 (CH_3_), 19.00 (CH_3_), 21.83 (CH_2_), 22.76 (CH_3_), 23.00 (CH_3_), 24.35 (CH_2_), 24.50 (CH_2_), 28.35 (CH_2_), 28.39 (CH), 28.57 (CH_2_), 28.66 (CH_2_), 32.40 (CH_2_), 35.11 (CH_2_), 35.38 (C), 35.88 (CH), 36.22 (CH), 36.66 (CH_2_), 39.92 (CH_2_), 40.47 (CH_2_), 43.01 (C), 45.51 (CH), 53.33 (CH), 56.68 (CH), 56.69 (CH), 108.09 (CH), 149.72 (C). MS (20°C): *m/z* (%) = 458 (100) [M^+^], 443 (16), 429 (16), 316 (10), 195 (10), 143 (68), 142 (41), 73 (14). HRMS: *m/z* [M^+^] calcd for C_30_H_54_OSi: 458.3944; found: 458.3930.Analysis calcd for C_30_H_54_OSi: C 78.53, H 11.86; found: C 78.59, H 11.91.

**4α-Fluoro-5α-cholestan-3-one (5)**

1-Fluoro-2,4,6-trimethylpyridinium triflate (77.2 mg, 267 µmol) was added to a solution of the trimethylsilyl enol ether (**4**) (123 mg, 267 µmol) in dichloromethane (3 ml). The reaction mixture was stirred for 60 h at r. t. under an argon amosphere and another portion of 1-fluoro-2,4,6-trimethylpyridinium triflate (38.6 mg, 133 µmol) was added. After stirring for 36 h, water (5 ml) and ether (10 ml) were added. The layers were separated and the aqueous layer was washed with ether three times. The combined organic layers were dried over sodium sulfate and the solvent was evaporated. Purification of the residue by flash chromatography (hexane/*tert*-butyl methyl ether, 10:1) on silica gel afforded 4α-fluoro-5α-cholestan-3-one (**5**) as colorless crystals, yield: 53.2 mg (44%), mp: 164-165°C. IR (ATR): ν = 2933, 2866, 2852, 1735, 1467, 1444, 1382, 1101, 1065, 1040, 890, 592 cm^–1^. ^1^H-NMR (500 MHz, CDCl_3_): δ = 0.66 (s, 3 H), 0.77 (m, 1 H), 0.849 (d, *J* = 6.6 Hz, 3 H), 0.853 (d, *J* = 6.6 Hz, 3 H), 0.89 (d, *J* = 6.5 Hz, 3 H), 0.95-1.17 (m, 8 H), 1.08 (s, 3 H), 1.20-1.43 (m, 9 H), 1.45-1.66 (m, 4 H), 1.75-1.86 (m, 2 H), 1.88-1.92 (m, 1 H), 1.96-2.05 (m, 2 H), 2.45 (m, 2 H), 4.72 (dd, *J* = 49.2, 12.3 Hz, 1 H). ^13^C-NMR and DEPT (125 MHz, CDCl_3_): δ = 12.04 (CH_3_), 12.91 (CH_3_), 18.64 (CH_3_), 21.12 (CH_2_), 22.54 (CH_3_), 22.80 (CH_3_), 23.65 (CH_2_), 23.80 (CH_2_), 24.17 (CH_2_), 28.00 (CH), 28.21 (CH_2_), 30.88 (CH_2_), 34.97 (CH), 35.76 (CH), 36.10 (CH_2_), 36.74 (CH_2_), 37.56 (d, ^3^*J*_C,F_ = 7.3 Hz, C), 38.74 (CH_2_), 39.48 (CH_2_), 39.71 (CH_2_), 42.50 (C), 52.96 (d, ^2^*J*_C,F_ = 15.9 Hz, CH), 53.89 (CH), 56.08 (CH), 56.19 (CH), 93.49 (d, ^1^*J*_C,F_ = 189.3 Hz, CH), 205.70 (d, ^2^*J*_C,F_ = 14.3 Hz, C=O). MS (20°C): *m/z* (%) = 404 (69) [M^+^], 389 (8), 250 (68), 249 (100), 235 (27). HRMS: *m/z* [M^+^] calcd for C_27_H_45_FO: 404.3454; found: 404.3475.

**4α-Fluoro-5α-cholestan-3β-ol (6)**

A 1M solution of lithium aluminum hydride (82 µl, 82 µmol) was added to a solution of 4α-fluoro-5α-cholestan-3-one (**5**) (27.7 mg, 68.0 µmol) in anhydrous THF (4 ml) at 0°C. After stirring for 24 h at 0°C under an argon atmosphere, water and then 10% HCl were added. The aqueous layer was extracted with ether three times. The combined organic layers were washed with a saturated solution of sodium bicarbonate and dried over sodium sulfate. Evaporation of the solvent and purification of the residue by flash chromatography (hexane/*tert*-butyl methyl ether, 5:1) on silica gel provided 4α-fluoro-5α-cholestan-3β-ol (**6**) as colorless crystals, yield: 11.8 mg (43%), mp: 99-105°C. IR (ATR): ν = 3566, 3337, 2932, 2866, 2849, 1467, 1382, 1163, 1145, 1081, 1059, 1024, 977, 929, 890, 613, 568 cm^–1^. ^1^H-NMR (500 MHz, CDCl_3_): δ = 0.64 (s, 3 H), 0.70 (m, 1 H), 0.82 (s, 3 H), 0.847 (d, *J* = 6.6 Hz, 3 H), 0.852 (d, *J* = 6.6 Hz, 3 H), 0.88 (d, *J* = 6.5 Hz, 3 H), 0.93-1.15 (m, 9 H), 1.18-1.37 (m, 9 H), 1.42-1.59 (m, 4 H), 1.67-1.77 (m, 2 H), 1.78-1.84 (m, 2 H), 1.86-1.91 (m, 1 H), 1.96 (dt, *J* = 12.6, 3.3 Hz, 1 H), 2.31 (br s, 1 H), 3.64 (m, 1 H), 4.16 (ddd, *J* = 52.6, 10.3, 8.6 Hz, 1 H). ^13^C-NMR and DEPT (125 MHz, CDCl_3_): δ = 12.05 (CH_3_), 13.37 (CH_3_), 18.65 (CH_3_), 20.89 (CH_2_), 22.34 (d, ^3^*J*_C,F_ = 3.6 Hz, CH_2_), 22.55 (CH_3_), 22.80 (CH_3_), 23.81 (CH_2_), 24.16 (CH_2_), 27.48 (d, ^3^*J*_C,F_ = 7.8 Hz, CH_2_), 28.00 (CH), 28.22 (CH_2_), 31.10 (CH_2_), 35.01 (CH), 35.77 (CH), 35.83 (CH_2_), 36.13 (CH_2_), 37.76 (d, ^3^*J*_C,F_ = 7.8 Hz, C), 39.49 (CH_2_), 39.86 (CH_2_), 42.50 (C), 49.09 (d, ^2^*J*_C,F_ = 15.0 Hz, CH), 54.27 (CH), 56.23 (CH), 56.31 (CH), 74.23 (d, ^2^*J*_C,F_ = 18.6 Hz, CH), 97.99 (d, ^1^*J*_C,F_ = 170.7 Hz, CH). MS (100°C): *m/z* (%) = 406 (48) [M^+^], 391 (17), 252 (50), 251 (100). HRMS: *m/z* [M^+^] calcd for C_27_H_47_FO: 406.3611; found: 406.3643. Analysis calcd for C_27_H_47_FO: C 79.74, H 11.65; found: C 79.89, H 11.47.

1. Stork, G., Rosen, P., and Goldman, N.L. (1961). The α-Alkylation of Enolates from the Lithium-Ammonia Reduction of α,β-Unsaturated Ketones. J. Am. Chem. Soc. *83*, 2965–2966.
2. Stork, G., and Darling, S.D. (1964). The Stereochemistry of the Lithium–Ammonia Reduction of α,β-Unsaturated Ketones. J. Am. Chem. Soc. *86*, 1761–1768.
3. Stork, G., Rosen, P., Goldman, N., Coombs, R.V., and Tsuji, J. (1965). Alkylation and Carbonation of Ketones by Trapping the Enolates from Reduction of α,β-Unsaturated Ketones. J. Am. Chem. Soc. *87*, 275–286.
4. Mazur, Y., and Sondheimer, F. (1958). Synthesis and Reactions of Ring A Methylated Saturated Steroids. J. Am. Chem. Soc. *80*, 5220–5229.
5. Mattern, G., Albrecht, P., and Ourisson, G. (1970). 4-Methyl-sterols and Sterols in Messel Shale (Eocene). Chem. Commun. 1570–1571.
6. Kokke, W.C.M.C., Fenical, W., and Djerassi, C. (1981). Sterols With Unusual Nuclear Unsaturation from Three Cultured Marine Dinoflaggelates. Phytochemistry *20*, 127–134.
7. Kokke, W.C.M.C., Bohlin, L., Fenical, W., and Djerassi, C. (1982). Novel Dinoflagellate 4α-Methylated Sterols from Four Caribbean Gorgonians. Phytochemistry *21*, 881–887.
8. Kokke, W.C.M.C., Fenical, W., and Djerassi, C. (1982). Sterols of the Cultured Dinoflaggelate *Pyrocystis lunula*. Steroids *40*, 307–318.
9. Tam Ha, T.B., Kokke, W.C.M.C., and Djerassi, C. (1982). Minor Sterols of Marine Invertebrates 37. Isolation of Novel Coprostanols and 4α-Methyl Sterols from the Tunicate *Ascidia nigra*. Steroids *40*, 433–453.
10. Dow, W.C., Gebreyesus, T., Popov, S., Carlson, R.M.K., and Djerassi, C. (1983). Marine 4-Methyl Sterols: Synthesis of C-24 Epimers of 4α,24-Dimethyl-5α-cholestan-3β-ol and 360 MHz ^1^HNMR Comparisons to the Natural Product from *Plexaura homomalla*. Steroids *42*, 217–230.
11. Harvey, H.R., Bradshaw, S.A., O'Hara, S.C.M., Eglington, G., and Corner, E.D.S. (1988). Lipid Composition of the Marine Dinoflagellate *Scrippsiella trochoidea*. Phytochemistry *27*, 1723–1729.
12. Stork, G., and Singh, J. (1974). Regiospecific Michael Reactions in Aprotic Solvents with α-Silylated Electrophilic Olefins. Application to Annelation Reactions. J. Am. Chem. Soc. *96*, 6181–6182.
13. Umemoto, T., Fukami, S., Tomizawa, G., Harasawa, K., Kawada, K., and Tomita, K. (1990). Power and Structure-Variable Fluorinating Agents. The *N*-Fluoropyridinium Salt System. J. Am. Chem. Soc. *112*, 8563–8575.

**Legend to supplementary figure:** TLC analysis reveals no gross level changes of cholesterol metabolism in wild type (lane 1, N2), null mutant *daf-12* (lane 2) or double null mutant *daf-9 daf-12* strains (lane 3). CE = cholesteryl esters, mS = methylated sterols (lophenol, 4-methylcholestenol), nmS = non-methylated sterols (cholesterol, 7-dehydrocholesterol, lathosterol).
